# Effect of Chitosan and bioactive glass nanomaterials as intracanal medicaments on TGF-β1 release from intraradicular dentin

**DOI:** 10.1186/s12903-025-07018-7

**Published:** 2025-10-16

**Authors:** Sarah Salah Hashem, Mohammed M. Khalefa, Mahmoud Hassan Mohamed, Hemat M. ELSheikh, Fatma Abd El-Rahman Taher

**Affiliations:** 1 Endodontist at Al-Zahraa University Hospital, Cairo, Egypt; 2 Professor of Endodontic, Faculty of Dental Medicine for Girls, Al-Azhar University, Cairo, Egypt; 3 Professor of Endodontic, Faculty of Dental Medicine, Al-Azhar University, Assuit Branch, Assuit, Egypt; 4 Professor of Endodontic, Faculty of Oral and Dental Medicine, Miser University of Science and Technology, Cairo, Egypt; 5 Associate Professor of Endodontic, Faculty of Dental Medicine for Girls, Al-Azhar University, Cairo, Egypt; 6 Professor of Chemistry, Faculty of Science, Al-Azhar University (Girls), Cairo, Egypt; 7 Head of Al-Azhar Nano-technology Incubator, Al-Azhar University, Cairo, Egypt

**Keywords:** Intracanal medicaments, Nanomaterials, Growth factors, Regenerative endodontics, Chitosan, Bioactive glass

## Abstract

**Aim:**

This study aimed to assess the influence of Bioactive glass nanoparticle (BAG-np) and Chitosan nanoparticle (CSNP) as intracanal medications compared with triple antibiotic paste (TAP) on the release of TGF-β1 from radicular dentin via enzyme-linked immunosorbent assay (ELISA).

**Methods:**

Forty dentin discs (1-mm thick) were horizontally cut from roots of 40 teeth.The samples were submerged in 20 ml of 1.5% NaOCL, followed by submersion in 20 ml 17% of EDTA, after which they were distributed equally into 2 experimental groups (I, II) and 2 control groups (III, IV) (10 samples each). Group I: (BAG-np), group II: (CSNP), group III: (TAP) (positive control) and group IV: nonmedicated (negative control). After 3 weeks, the medications were removed via Phosphate buffer saline (PBS), and the samples were subsequently submerged in PBS for 24 h at 37 °C. The medium from the samples was collected and used to measure the TGF-β1 concentration via ELISA.

**Results:**

There was a statistically significant difference between the BAG-np, CSNP, and nonmedicated groups compared to the TAP group (P-value < 0.05). The highest value of released (TGF-β1) was recorded in the CSNP group, followed by the BAG-np group, then the nonmedicated group, whereas the lowest value was recorded in the TAP group.

**Conclusion:**

BAG-np and CSNP induce the release of TGF-β1after irrigation with 1.5% NaOCl and 17%EDTA, significantly greater than TAP. Accordingly, BAG-np and CSNP may be used as alternatives to TAP in regenerative endodontic procedures.

## Introduction

 The succession of microbial infections that invade the pulp system leads to irreversible pulpitis and pulp necrosis, resulting in periapical periodontitis [1]. Root canal treatment (RCT) is the true management method for the necrotic pulp of mature teeth; however, in immature teeth with incomplete root formation, open apices and thin dentin walls, the RCT seems difficult and challenging because of their vulnerability to injury and root fracture [2]. Apexification is a treatment modality for immature teeth that involves either tri-calcium silicate matrix placement at the root apex or the use of calcium hydroxide to prompt the formation of apical barrier; however, these techniques do not induce completion of the root length to width [3].

Regenerative endodontics is a biological approach designed to reconstruct vital tissue in the root canal system [4]. This revascularization process in immature pulpless teeth encourages the completion of root formation and apical closure [5]. Regenerative therapies in endodontics depend mainly on tissue engineering which need the presence of main triad of bioactive growth factors (GFs), biomimetic scaffolds, and stem cells [6].

Stem cells (SCs) are self-renewable and can differentiate into manifold tissue lineages, which constitute the fundamental component of the tissue engineering process [7]. The central niches that contribute to SCs revitalization include dental pulp stem cells (DPSCs), which markedly decrease after pulp necrosis, and stem cells from apical papilla (SCAPs) which share (DPSCs) as a cell source for initial root construction; however, after pulp necrosis, important SC sources such as hematopoietic stem cells (HSCs), human periodontal ligament stem cells (PDLSCs) which reside between the cementum and the bone around the root, and human bone marrow stromal stem cells (BMSSCs) can be used in regenerative treatment [7, 8]. Stem cell destiny depends mainly on GFs, which are polypeptide growth-regulatory molecules that control cell migration, and stimulate cell proliferation and differentiation [9].These progenitor chemoattractants include transforming growth factor β1 (TGF-β1), which is a pleiotropic molecule and a member of the TGF-β family and is involved in signaling procedures and the recruitment of SCs, which are important for inducing regeneration and stimulating pulpal tissue repair [10, 11].

The bioactive growth factors are secreted by pulp fibroblasts and odontoblasts during tooth development and then stored in the dentin matrix, which acts as the main reservoir for such bioactive components, which can be emitted through tissue demineralization during dental caries and restoration procedures to mediate the dentin repair process [12, 13]. TGF-β1 entrapped in dentin can be reactivated during the process of root canal disinfection via demineralizing chemicals, and the liberation of these bioactive gradients can stimulate the survival of preexisting odontoblasts and promote the differentiation of SCs, which is the base of the revitalization progression [13].

Proper disinfection of the root canal system is essential for achieving the desired outcome in the endodontic regeneration protocol, because SCs proliferation can occur only in a bacteria free biological environment in the root canal space [14]. However, the complex root canal anatomy prevents the routine chemo-mechanical preparation from completely disinfecting this trajectory; thus, the use of intracanal medicaments with antimicrobial properties is mandatory to create a sterile medium for pulp tissue reconstruction [15].

Many therapeutic agents and chemicals have been proposed as disinfectants and preservatives for inducing dental SCs due to their bacteriostatic and bactericidal properties. Triple antibiotic paste (TAP) is one of the most commonly used endodontic medicaments, consisting of minocycline, metronidazole, and ciprofloxacin. This medicament can efficiently get rid of intracanal microorganisms and provides an appropriate environment for stem cells binding and differentiation [16]. Since chemical therapeutics formulations have potential side effects and safety concerns, researchers have recently focused on natural products that may have greater antimicrobial activity, biocompatibility, and anti-inflammatory and antioxidant effects [17].

The new revolution of nanotechnology, with its promising future in different fields, including dentistry in general and endodontics in particular. The nanoparticles are known as microscopic particles with sizes ranging from 1 to 100 nm and at least one dimension. These criteria provide nanoparticles with unique properties, such as stellar surface area, charge density, and remarkable cell communication, that improve their antimicrobial activity, which effectively disrupts bacterial biofilm in the root canal system [18, 19].

Chitosan (CS) is a natural component obtained from crustacean exoskeletons through alkaline deacetylation. It has a chelating effect on interradicular dentin, an antimicrobial influence on a broad range of bacteria and fungi, is highly biocompatible and enhances root canal dentin degradation by collagenase, which improves fracture resistance [20]. The integration of Chitosan nanoparticles enhances their biocompatibility to be more convenient for revitalization and the employment of tissue engineering [21]. Bioactive glass (BAG), which consists of different concentrations of SiO2, Na2O, CaO2, and P2O5, has antimicrobial effects attributed to its high pH, Ca/P precipitation and high osmotic pressure [22]. It has controlled degradability and the ability to motivate formation of new tissue, making it a promising material for tissue engineering [23].

Intracanal medicaments can affect the liberation of GFs from radicular dentin, which may consequently induce the proliferation and differentiation of SCs [9, 24]. Therefore, this study investigated the effects of Bioactive glass nanoparticles (BAG-np), Chitosan nanoparticles (CSNP), and TAP on the liberation of TGF-β1 from radicular dentin. The null hypothesis posits that the examined groups have a similar effect on TGF-β1 liberation.

## Materials and methods

### Ethics approval and consent to participate

The study was operated after acquiring the approval of the research ethics committee (REC) of the Faculty of Dental Medicine, Al-Azhar University, with the primary code (P-EN-21-04) and the final code (REC-PD-25-02a). The study was performed on extracted teeth. The extracted human teeth used were obtained anonymously from dental clinics, without any identifiable patient data. Therefore, no specific informed consent was required. The study was conducted in accordance with institutional ethical guidelines for the use of biological tissues.

### Sample size

In order to operate a statistical test of the null hypothesis “which presumes that the release of TGF-β1 from radicular dentin would be the same” a power analysis was created using BAG-np, CSNP and TAP. The expected sample size (n) was determined to be at least (9 root dentin discs) instances by utilizing an alpha level of (0.05) and a beta of (0.2), meaning power = 80% and an effect size (d) of (15) was computed based on the findings of Ghonimy et al. [25]. The G*Power version 3.1.9.7 software (Heinrich Heine University, Düsseldorf, Germany).

### Preparation of bioactive glass nanoparticles

Bioactive glass 45S5 was obtained from a commercial trader (PerioglassTM, US Biomaterials Corp., Alachua, FL, USA). As stated by the manufacturer, this material contains 45 wt% SiO2, 6 wt% P2O5, 24.5 wt% CaO, and 24.5 wt% Na2O. Preparation of BAG-nps were accomplished using the sol-gel technique (Nano Gate, Cairo, Egypt). The oxide composition was produced using silicon and phosphorus alkoxides, along with sodium salt (sodium hydroxide) and calcium salt (calcium hydroxide). Deionized water and ethyl alcohol were used as solvents. The gel was prepared at 70 °C and a pH of approximately 2, aged for one week to finish the reaction, and then treated with heat at a maximum temperature of 800 °C [26].

### Preparation of Chitosan nanoparticles

Chitosan nanoparticles were prepared at the Faculty of Science, Al-Azhar University. The formulation of CSNPs was based on the ionotropic gelation of Chitosan (CS) with tripolyphosphate (TPP) [27]. Chitosan with Low molecular weight (Nano Gate, Cairo, Egypt) and a degree of deacetylation between 75% and 85% was disintegrated in 1% (v/v) acetic acid at a concentration of 2% (w/v). TPP (EMD Millipore, Billerica, MA, USA) was disintegrated in sterile distilled water to form a 0.5% (w/v) solution. CSNPs were produced upon adding the TPP solution to the CS solution in acetic acid at a ratio of 1:4 (TPP: CS) by using a magnetic stirrer at 1000 rpm at room temperature.

The separation of CSNPs was performed via centrifugation at 60,000 rpm for 15 min. The supernatant was removed, and the CSNPs were rinsed with deionized water. Then the mixture was dried by freezing. After drying, the mixture was crushed by using a manual grinder until a homogeneous powder was obtained.

### Characterization of Chitosan nanoparticles

**Fig. 1:**
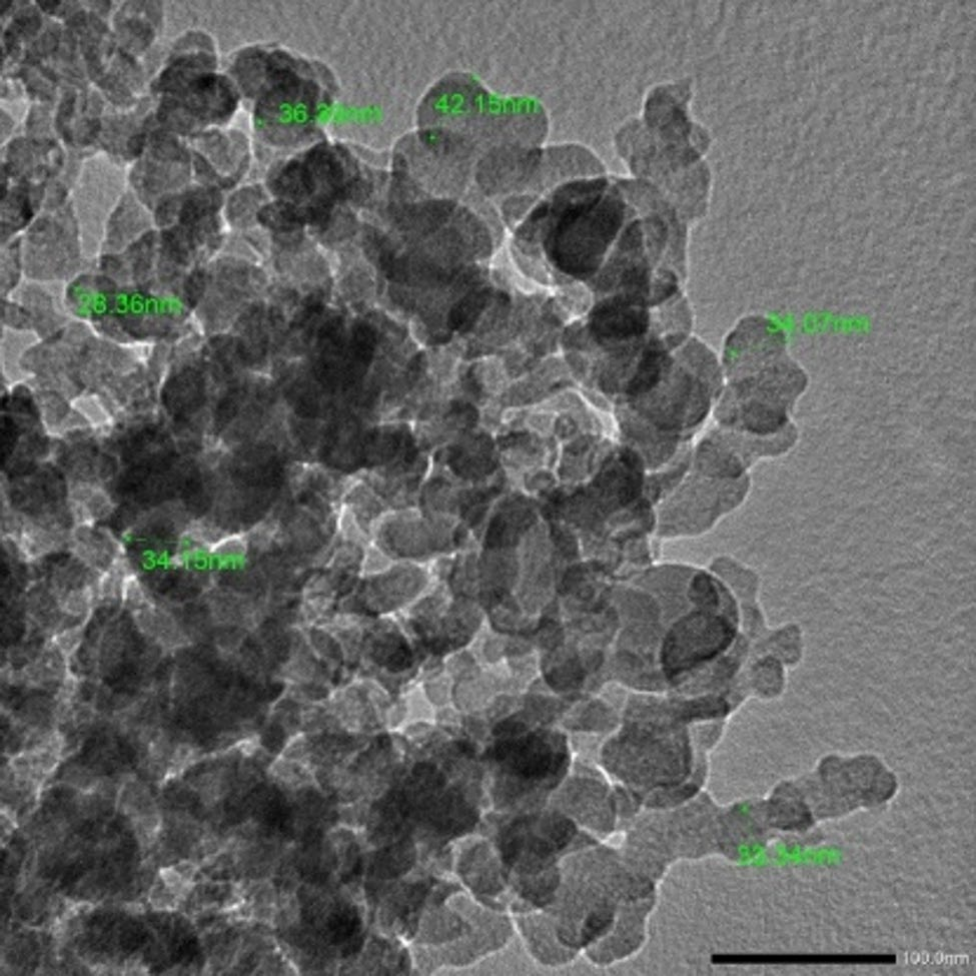
TEM image showing CSNPs

Chitosan nanoparticles were examined by the aid of high-resolution transmission electron microscope (TEM) (JEM-2100 plus, JEOL ltd., Tokyo, Japan) at faculty of science, Al – Azhar university, at an accelerating voltage of 200KV for the detection of the size and shape of the prepared nanoparticles. The examination revealed the creation of identical spherical-shaped particles, with sizes of less than 50 nm (Fig. 1). The overall charge of the prepared nanoparticles was determined by assessing the zeta potential via the dynamic light scattering technique among a zeta potential analyzer (Particle Sizing Systems, Santa Barbara, California, USA) [27].

### Preparation of triple antibiotic paste

Triple antibiotic paste consisting of Metronidazole 500 mg tablets (Flagyl 500 mg, Aventis, Cairo, Egypt), ciprofloxacin 250 mg tablets (Ciprocin 250 mg, EPICO, Cairo, Egypt) and doxycycline 100 mg capsules (Vibramycin, Pfizer, Cairo, Egypt). The Doxycycline capsule content, a tablet of Metronidazole and a tablet of Ciprofloxacin were crushed and ground into a homogenous powder in manual grinder [28].

### Sample selection and Preparation of root discs

Forty sound and completely developed human premolars were obtained anonymously from dental clinics, without any identifiable patient data. The teeth were visually examined and proven by X-ray to ensure that the root canals were Vertucci type I.

The freshly extracted teeth were cleansed with phosphate-buffered saline (PBS), and the periodontal tissues were removed by scraping the root surface with a scalpel blade and then kept in 0.5% Chloramine-T trihydrate solution at 4 °C for up to 4 months. Before tooth preparation, the storage solution was changed to distilled water and left for 24 h at room temperature. The crowns of the teeth were cut off using a double-faced diamond disc, and the pulp tissue was extirpated from the root canal lumen with hand K-files size 15 (Mani Inc., Japan). Root dentin discs, roughly 1 mm thick, were split perpendicularly to the long axis from the middle third of the root using the Isomet 4000 microsaw (Buehler, USA) with a water coolant. One disc was gained from each root [25, 29].

### Treatment of root dentin discs

Forty root dentin discs were submerged in 20 ml of 1.5% sodium hypochlorite (NaOCl) and incubated at 37 °C for 5 min. Then they were submerged in 20 ml of 17% ethylenediaminetetraacetic acid (EDTA) and once more kept in the incubator at 37 °C for another 5 min. The discs were divided equally into 2 experimental groups (I, II) according to the type of intracanal medication and 2 control groups (III, IV) (10 samples each). Group I: BAG-np, group II: CSNP, group III: TAP (positive control group) and group IV: Nonmedicated (negative control group).

All the medications were prepared at room temperature. BAG-np powder was mixed with distilled water at a ratio of 1:1 wt/vol to get a homogeneous, smooth, creamy paste [25]. A total of 1.8 g of CSNPs powder was mixed with 0.5 ml of 1% acetic acid to obtain a creamy paste [30]. Equal portions of Doxycycline, Metronidazole, and Ciprofloxacin (at a ratio of 1:1:1) were mixed with saline to form TAP mixture with a final concentration of 5 mg/ml [31].

**Fig. 2:**
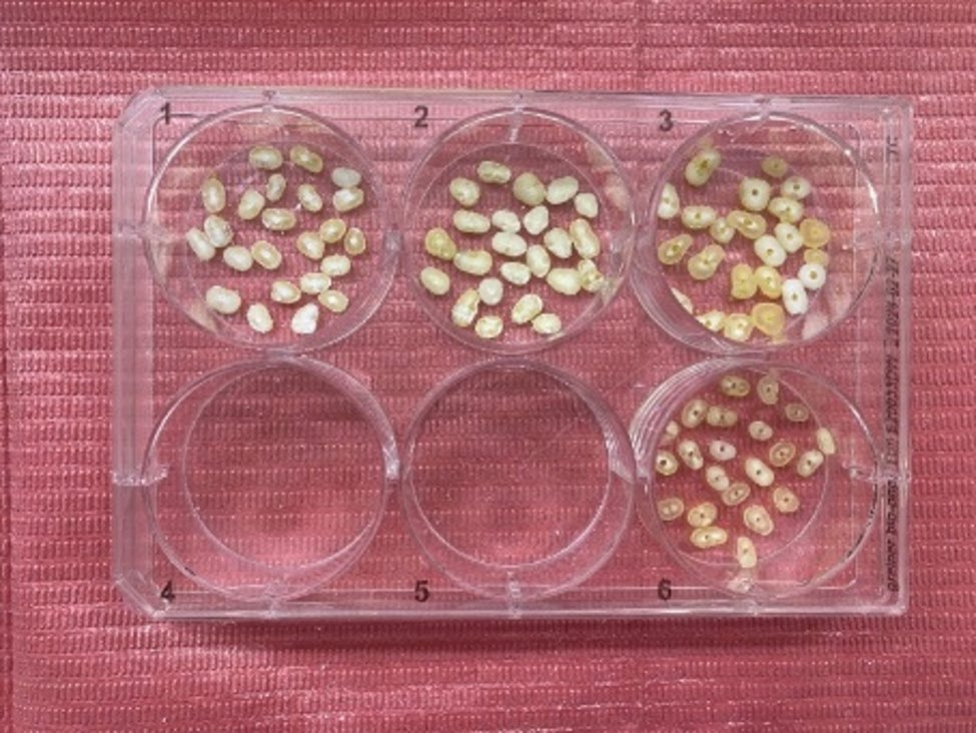
A photograph showing root dentin discs filled with different intracanal medications and a group of empty discs (negative control)

**Fig. 3:**
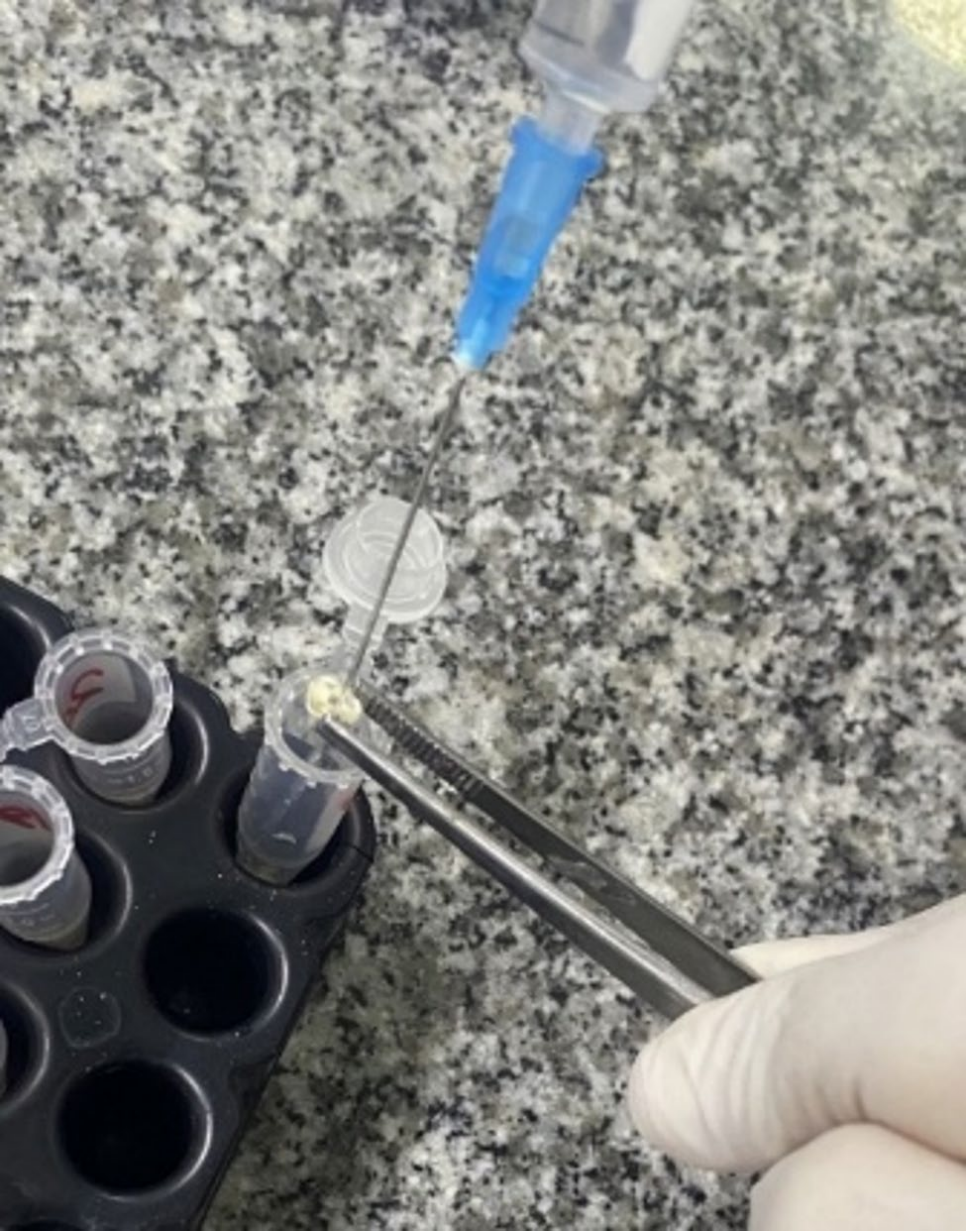
A photograph showing the washing of intracanal medication from root dentin discs with PBS

All the medications were applied with a metal spatula to the canal lumen inside the dentin discs, while dentin discs of the negative control group remained empty (Fig. 2). All dentin discs were subsequently stored at 100% relative humidity and 37 °C for 3 weeks. After this period, the medications were removed by using PBS (Fig. 3), and then the root discs were immersed in PBS for 24 h at 37 °C. The root discs of the negative control group were also placed in PBS after the storage period.

### Quantification of released TGF-β1

The medium from the samples was collected and then filtered to measure the amount of TGF-β1 released into the collected medium using the enzyme-linked immunosorbent assay (ELISA) following the protocol applied by the manufacturer (Human TGF-β1 ELISA Kit, Elabscience Biotechnology, USA). The resulting absorption values were consistent with those of the standards delivered with the ELISA kit. Each sample was assayed in triplicate.

## Results

**Fig. 4:**
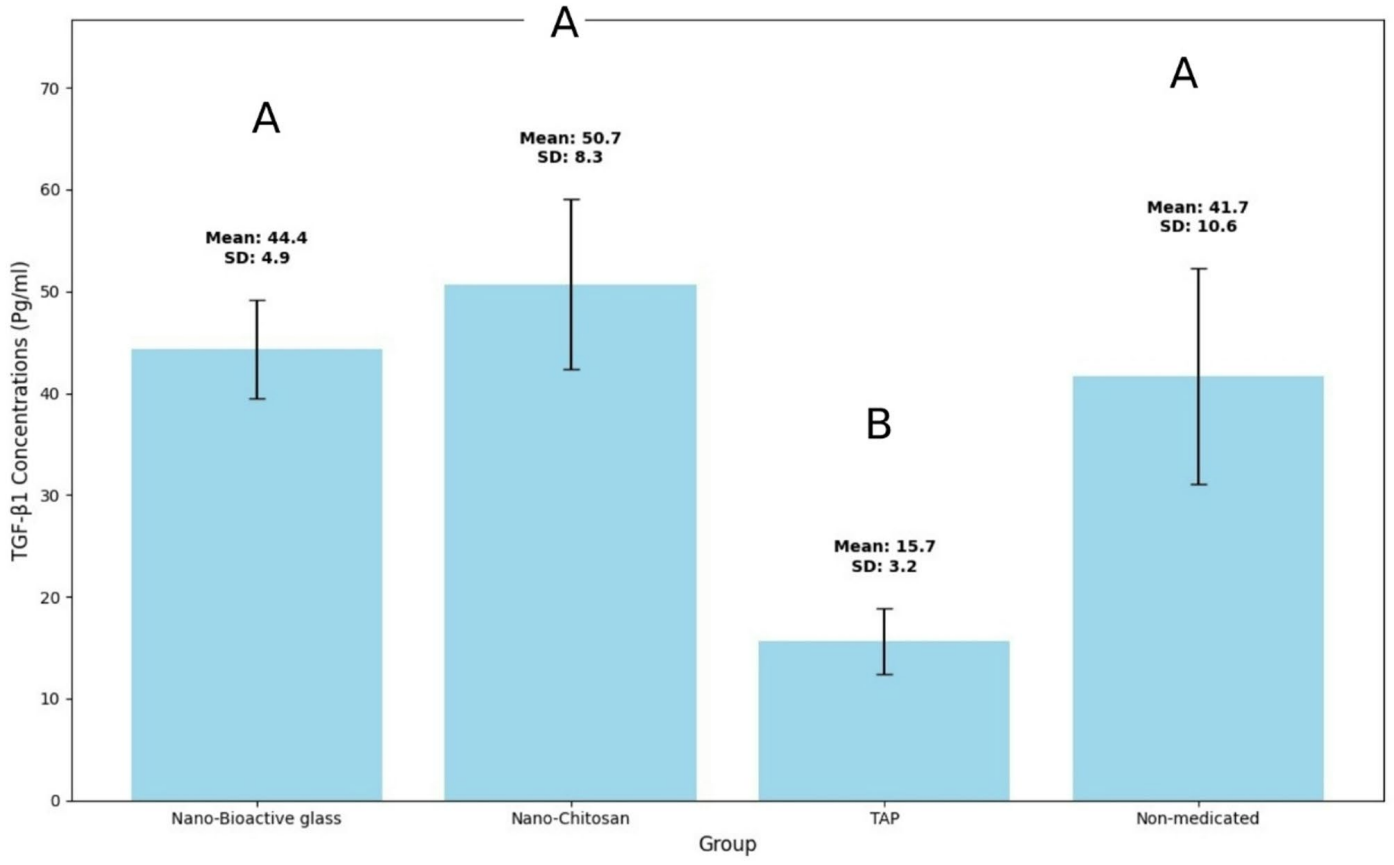
A bar graph showing mean values and standard deviation of TGF-β1 concentration (Pg/ml) results in different groups. Different letters indicate statistically significant difference among groups (*P* ≤ 0.05)

TGF-β1 concentration data were collected, and the statistical analysis was done utilizing IBM SPSS Statistics for Windows (Version 23.0. Armonk, NY: IBM Corp). To indicate any statistical difference among the tested groups, a (*P* ≤ 0.05) significance level was established. The Shapiro- Wilk and Kolmogorov Smirnov tests were used to test the continuous variables for normality. One-way analysis of variance (ANOVA) test and Post-hoc Tukey test were used to compare the mean values and standard deviation of TGF-β1 concentration (pg/ml). Descriptive statistics of TGF-β1 concentration values for each group are expressed in (Table 1) and (Fig. 4).

**Fig. 5:**
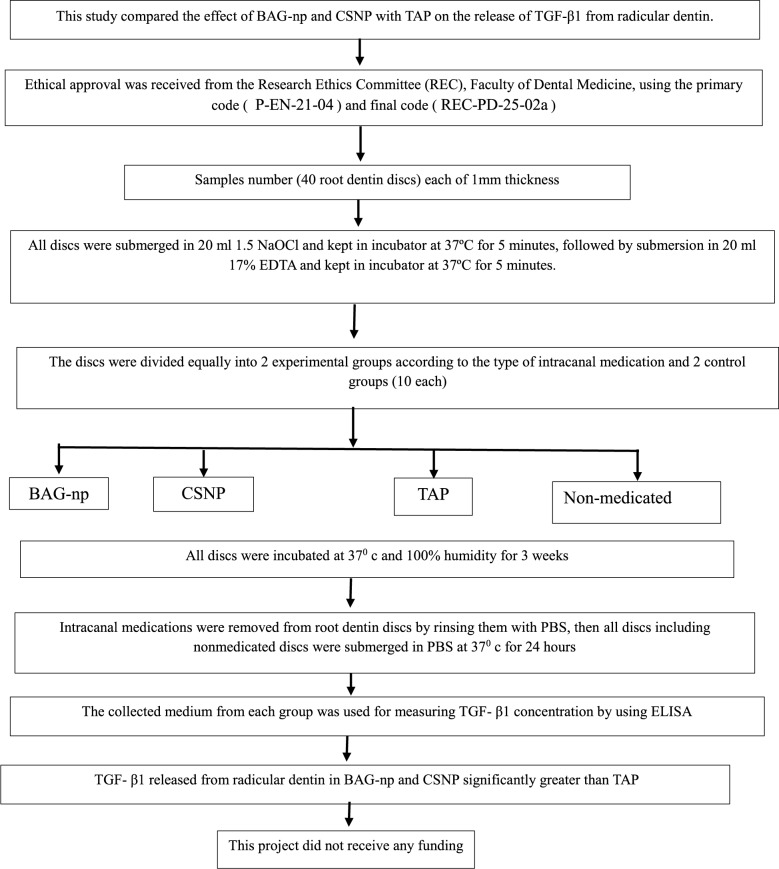
Flowchart showing research design and results

TGF-β1 was detected in all groups, with the highest mean concentration value determined in CSNP group (50.7 pg/ml ± 4.9), followed by BAG-np group (44.4 ± 4.9), nonmedicated group (41.7 pg/ml ± 10.6) and TAP group (15.7 pg/ml ± 3.2) respectively. There was no statistically significant difference among the BAG-np, CSNP, and non-medicated groups. However, a statistically significant difference was found between the BAG-np, CSNP, and non-medicated groups compared to the TAP group. The research design flowchart is shown in Fig. 5.

**Table 1:** The mean values, standard deviation (SD) and P-value of TGF-β1 concentrations (Pg/ml) for all tested groups

Group (*n* = 10)	Mean	SD	*P*-value
Group I (BAG-np)	44.4^a^	4.9	< 0.001^*^
Group II (CSNP)	50.7^a^	8.3	
Group III: Positive control group (TAP)	15.7^b^	3.2	
Group IV: Negative control group (Nonmedicated)	41.7^a^	10.6	

* Significant at *P* ≤ 0.05, Different superscripts indicate statistically significant differences between groups

## Discussion

Regenerative endodontics is a biological strategy that stimulates the emergence of a new structure that replaces vital tissue and is currently used for the management of immature pulpless teeth, which aims to reinstate the pulp’s ability to reform root tertiary dentin [4]. Chemical disinfection, which is a critical step of the treatment procedure, has attracted special interest as it has the potential to release a range of dentin matrix components, including GFs, the major regulatory stem cell growth molecules [32]. According to the AAE, Calcium Hydroxide (CH) or a low concentration of TAP has been indicated for cases of REP because this disinfection method has been clinically and radiographically successful [24].

In this study, TAP was selected for its superior antibacterial effect against E. faecalis compared to CH [33]. The antibacterial efficacy of CH directly lies within the diffusion of alkaline hydroxyl ions. After a week of CH introduction inside the canal, pH reaches its maximum values and then begins to drop. As the pH falls, the residual bacteria may regrow in the canals treated with CH [34], while TAP exhibits its antibacterial efficacy by targeting the diverse and complex microbial communities within the root canal system. The synergistic action of ciprofloxacin, metronidazole, and minocycline is key to its effectiveness, as each component targets different microbial pathways. Ciprofloxacin, a fluoroquinolone, inhibits bacterial DNA gyrase and topoisomerase IV, preventing DNA replication and transcription, which is particularly effective against Gram-negative bacteria. Metronidazole, a nitroimidazole derivative, disrupts anaerobic bacterial DNA, making it indispensable for targeting anaerobic pathogens often present in endodontic infections. Minocycline, a tetracycline antibiotic, inhibits protein synthesis by binding to the 30 S ribosomal subunit, showing efficacy against Gram-positive bacteria. This broad-spectrum activity ensures that TAP addresses the polymicrobial nature of necrotic pulp and periapical infections, creating an environment suitable for tissue healing [35].

Nanomaterials have been used in endodontics and can be presented in different materials as intracanal medicaments. The nanoform of materials increases the surface area and promotes better penetration through root canal dentin, allowing the acquisition of the most valuable effect of the medicaments, which can improve the release of the bioactive component at higher concentrations into the root canal [36]. Thus, the current study was directed to evaluate the effectiveness of BAG-np and CSNP used as intracanal medications on TGF-β1 release from radicular dentin compared to the traditionally used TAP and the null hypothesis was rejected.

TGF-β1 was chosen for evaluation in this study because growth factors are thought to help in creating an ideal environment for regeneration. Various growth factors have already been integrated into the root canal system to enhance the formation of dental pulp-like tissue and promote dentinogenesis. TGF-β1, a member of the transforming growth factor-β family, is a multifunctional protein that is abundant and widely distributed. It serves as a key growth factor involved in the repair of pulp tissue and the process of dentinogenesis. TGF-β1 plays an important role in regulating collagen turnover, as well as the differentiation and proliferation of dental pulp cells [37].

In this study, TGF-β1 was detected and evaluated using ELISA, which is a diversified enzyme immunoassay (EIA) technique that uses the catalytic properties of enzymes to quantify immunologic reactions; thus, this method can measure substances at very low concentrations with minimal risk of interference [38]. The ELISA kit used in this study employs the Sandwich-ELISA principle. In this method, a microtiter plate is precoated with an antibody (human TGF-βI), which enables the capture and precise quantification of specific antigens from the prepared solution. This type of ELISA offers several advantages: it has high sensitivity, approximately 2 to 5 times greater than that of direct or indirect ELISAs; high specificity, as it uses only two antibodies to identify the antigen; and flexibility, allowing for the use of either direct or indirect techniques. Additionally, it is particularly suited for analyzing complex samples, as there is no need to purify the antigen prior to the assay, while still maintaining high specificity and sensitivity [39].

Phosphate bufferred saline is a storage medium with a pH of 7.2–7.4 that contains Na^+^, PO_4_^−3^ and Cl^−^ ions. It preserves the electrolyte imbalance in the solution while keeping the pH constant, thus, it could be used to store dentin discs without affecting their properties. Therefore, PBS was chosen for immersion of the dentin discs before performing ELISA test to avoid any interference with the expected results [40].

The organic component of dentin is composed mainly of type 1 collagen and dentin-specific proteins, which contain a wide range of entrapped transforming growth factors that play a critical role in endodontic regeneration as improving factors for cell differentiation and proliferation [41]. The clinical recommendations for regenerative procedures stated the use of 1.5–3% NaOCl solution followed by chelating agent (17% EDTA) as a standard irrigation protocol. The chelating agent will demineralize the dentine allowing the liberation of embedded bioactive components. The use of intracanal medicaments is expected to influence TGF release. Consequently, both the irrigating solutions and the intracanal dressing applied during regenerative procedures will affect its release [4].

In the present study, all tested groups demonstrated the release of TGF-β1. Although the irrigation protocol remained the same, the intracanal medicament used resulted in varying levels of TGF-β1. In the negative control group, where the root canal was conditioned with 17% EDTA alone and no dressing was used, the release of TGF-β1 was lower than that in the two tested groups, which were treated with BAG-np and CSNP, but the difference was not significant. This can be interpreted on the basis that TGF-β1 is superficially located in the dentin matrix and its binding is strongly stable in hydroxyapatite through the chondroitin sulfate chain, which has an affinity for calcium ions. When exposed to a chelating agent, its binding to the matrix can be readily disrupted, and it can be easily separated [42]. The EDTA chelation mechanism depends on destabilization of the inorganic dentin fraction; therefore, proteins and bioactive factors found in apatite crystals are released. This finding was in accordance with the well-documented ability of EDTA to release signaling molecules through the demineralization property of the dentin extracellular matrix [43]. However, it was antagonist to another study which reported that the control group treated with EDTA alone without using medicaments indicated higher TGF-β1 release than the groups treated with calcium silicate-based temporary intracanal medicament (Bio-C Temp) and calcium hydroxide-based intracanal medicaments (Ultracal XS) did; this may be related to that both of the tested medicaments used in the previous study typically raise the pH up to 10.79 (Bio-C Temp) and 11.01 (Ultracal XS), which may induce the denaturation of dentin’s organic components and thus create superficial obliteration zones, limiting the release of growth factors that are entrapped in the deeper dentin layers [29].

The highest amount of TGF-β1 release was observed in group II, which was treated with CSNP. This was in accordance with studies reported that root canal dentin conditioned with CSNP showed high concentration of TGF-β1; however, they used it as irrigating solution [44, 45]. Clinically, intracanal medicaments are applied in the canal for longer contact periods with root canal dentin which is expected to magnify the effect. Chitosan is a natural hydrophilic biopolymer; its nanometric range has free hydroxyl groups with amino functional groups, which are responsible for promoting surface hydrophilicity, resembling glycosaminoglycans. The interaction between positively charged CS units in glucosamine and negatively charged dentin surfaces results in destabilization of hydroxyapatite and dentin chelation with subsequent TGF-β1 liberation [36, 46].

The bioactive glass nanoparticles as intracanal medicament represented the second highest release level of TGF-β1, with no significant difference between CSNP and negative control groups. Bioactive glasses have been used in the endodontic field in various treatment options, and can be used as intracanal disinfectant due to their antimicrobial properties, which related to their increasing pH. Also, BAG is highly biocompatible and has been shown to stimulate osteoconduction and osteoinduction; thus, it has been increasingly used in endodontic regeneration and tissue engineering [25, 47]. A recent study has shown that hydrogel medicaments loaded with BAG significantly enhance the mineralization of DPSCs while maintaining cytocompatibility, in comparison to antibiotic-loaded hydrogel medicaments used in regenerative endodontic treatment [48]. Moreover, in this study, it was discovered that the group treated with BAG-np exhibited a significantly higher release of TGF-β1 compared to the group treated with TAP. Bioactive glass may promote the precipitation of calcium phosphate and the formation of hydroxyapatite, which may allow growth factors to adsorb onto the dentin surface [49]. These finding highlights the promising potential of bioactive glass in promoting tissue regeneration.

The lowest level of TGF-β1 was observed in the positive control group treated with TAP, which was significantly different from the other groups. TAP containing metronidazole, ciprofloxacin and minocycline has been proposed as a root canal medicament aimed at achieving a relatively aseptic environment due to its antimicrobial properties. Even at low TAP concentrations, it has a drawback effect on the proliferative capability and mineralized matrix genesis of dental pulp and apical papilla cells. Minocycline, a component of TAP, causes tooth discoloration. Also, it was observed that TAP causes dentin demineralization and decreases dentin micro-hardness, which resulted from chemical changes in the superficial dentine structure [50, 51]. Another potential explanation may be related to the incomplete removal of TAP during root canal irrigation. A previous study demonstrated that TAP was not completely removed, despite the use of an irrigation activation technique [52]. The presence of residual TAP on the dentin surface may interfere with the release of growth factors by occluding dentinal tubules and sealing the dentin surface [29]. This interference and chemical changes in dentin tissue may compromise the overall effectiveness of TAP regarding growth factor release.

As for the limitations of the current study, CSNP paste contains acetic acid, which may demineralize dentin, resulting in an increase in the liberation of TGF-β1. The results of the current study could be considered as a starting point for future research on the biocompatibility and antibacterial effects of BAG-np and CSNP in comparison to TAP. Furthermore, it would be beneficial to compare the effect of BAG-np and CSNP on TGF-β1 release with that of calcium hydroxide.

## Conclusions

The results of this study suggest that BAG-np and CSNP as intracanal medicaments can promote the success of REPs since they revealed an increase in the release of TGF-β1 following their use after irrigation with 1.5% NaOCl and 17%EDTA. Also, they induce the release of TGF-β1 significantly greater than TAP. Accordingly, BAG-np and CSNP may be used as alternatives to TAP in REPs.

## Data Availability

All data used and/or analyzed during this research are available from the corresponding author on reasonable request.

## Abbreviations

RCT: Root canal treatment
GFs: Growth factors
SCs: Stem cells
DPSCs: Dental pulp stem cells
SCAPs: Stem cells from apical papilla
HSCs: Hematopoietic stem cells
PDLSCs: Periodontal ligament stem cells
BMSSCs: Bone marrow stromal stem cells
TGF-β1: Transforming growth factor β1
TAP: Triple antibiotic paste
CS: Chitosan
BAG: Bioactive glass
BAG-np: Bioactive glass nanoparticles
CSNP: Chitosan nanoparticles
TPP: Tripolyphosphate
TEM: Transmission electron microscope
PBS: Phosphate-buffered saline
NaOCl: Sodium hypochlorite
EDTA: Ethylenediaminetetraacetic acid
ELISA: Enzyme-linked immunosorbent assay
CH: Calcium hydroxide
